# Distribution Network for the Last Mile of Cross-Border E-business in a Smart City at Emerging Market in Response to COVID-19: A Key Node Analysis Based on a Vision of Fourth Party Logistics

**DOI:** 10.3389/fpubh.2021.765087

**Published:** 2021-10-11

**Authors:** Da Huo, Xiaotao Zhang, Yinghui Cai, Ken Hung

**Affiliations:** ^1^School of International Trade and Economics, Central University of Finance and Economics, Beijing, China; ^2^School of Law, Beijing Institute of Technology, Beijing, China; ^3^A.R. Sanchez School of Business, Texas A&M International University, Laredo, TX, United States

**Keywords:** last mileage distribution, fourth party logistics, key node analysis, smart city in emerging market, cross-border E-business

## Abstract

This research studies the development of distribution networks for the last mile distribution for cross-border E-business based on a vision of fourth party logistics (4PL) in smart cities in emerging markets in response to COVID-19. This research analyzes the distribution centers of distribution companies in Beijing city using fuzzy cluster analysis as a case study of smart cities. The location decision for distribution centers to serve cross-border E-business is further analyzed by considering the local conditions of the distribution centers. The solutions to the location decisions for distribution centers in different cases are further visualized by 2-mode networks. The key nodes in the distribution network of the last mile for cross-border E-business are further studied based on fourth-party logistics by a immune algorithm. Cross-border E-business value creation based on the development of distribution networks using fourth-party logistics is further discussed. The location distribution of key nodes can spread from the downtown district to suburban areas as the coverage of the distribution network is expanded. This research can help managers and decision makers address the last mile distribution for cross-border E-business in smart cities in emerging markets based on a vision of fourth-party logistics in response to COVID-19.

## Introduction

Cross-border E-business can be developed by satisfying the specified and professional demands of customers, and the integration of services for enterprises, customers and supply chain management can be important. Value-adding services can be further upgraded by offering informational services (1). Cross-border E-businesses have transitioned their resource allocation from being material resource dependent to being information resource dependent, and they have thus become more knowledge driven and information intensive (2). Synergies across departments with different functions can help to improve service quality and operational efficiency in cross-border E-businesses in Beijing city (3).

The development of logistics systems can be important for the further development of cross-border E-businesses in smart cities. The development of logistics is an important measure that enhances the development of cross-border E-businesses in Beijing (4). The decisions of key node enterprises and improvements in their operational efficiency for cross-border E-business are important to developing competitiveness by further upgrading databases and information systems in the cross-border E-business arena. Fixed assets such as infrastructure and intangible assets such as technological know-how are important for the development of IT companies (5).

The development of cross-border E-businesses has extended from consumption to the supply chain, and intelligent logistics systems are further emphasized for structuring a well-developed cross-border E-business system in Beijing (6). Decisions regarding the allocation of resources in cross-border E-businesses are important for the development of an integrated cross-border E-business service system and for the competitive strength of Beijing in its involvement in the international cross-border E-business market. An cross-border E-business is an interactive system that integrates the flows of materials, capital, information and businesses, and logistics is an important part of this integrative system (7). Therefore, it is important to further analyze the distribution system of cross-border E-businesses and discuss the integration of distribution centers, aiming to offer more efficient services to cross-border E-business.

The distribution of commodities became a challenge to disease control during the COVID-19 pandemic. Channel management is important in online distribution systems to support the transformation of traditional businesses as they moved from offline to online in the COVID-19 period (8). It is important for emerging markets to develop business solutions in response to COVID-19 (9), and the distribution of products can be important to export business operators in the COVID-19 period (10). A smart system offers powerful support for cost efficiency and risk-adjusted performance (11), and fourth-party logistics can further improve the efficiency of the supply chain and promote the development of the social economy through strong resource integration and an optimized supply chain scheme (12). By integrating resources and advantages, fourth-party logistics aims to explore and leverage the collective competitive advantage and build a service portfolio characterized by management innovation awareness (13). Therefore, it is important to further develop the study of distribution networks based on a vision of fourth-party logistics in the last mile of cross-border E-businesses. A key node analysis of the distribution network offers further support to cross-border E-businesses responding to COVID-19 and can help to further employ a last mile distribution network in smart cities in emerging markets in the response to COVID-19.

## Literature Review

The development of online services was demanded in the COVID-19 period, and the new infrastructure of network services offers further support for the broader coverage of emerging online businesses (14). Distance management in COVID-19 is important to disease control and to the organized resumption of work and production (15). Distribution management in the COVID-19 period is important in disease control, and a stable supply of critical supplies is also important to support managerial work (16). The development of logistics strategies can be important for cross-border E-businesses (17). The operating environment is important for business solutions in emerging markets (18). The new generation of technology, including cloud computing, 5G system networks, and intelligent global systems, can help in the development of cross-border E-businesses based on the cutting-edge application of information technology and artificial intelligence, which offer further synergies to cross-border E-business operations. Such knowledge-driven synergies will motivate technological development and its applications (19). The development of technology can help further upgrade services for cross-border E-businesses. Support from big data and intelligent urban systems can also help by motivating cross-border E-businesses to adopt a greater service orientation.

Fourth-party logistics reduces costs through a relatively perfect integrated supply chain solution and further optimize technical solutions to bring value to the overall supply chain (20). By sharing social information resources, fourth-party logistics integrates third-party logistics resources to provide integrated logistics services to enterprises (21). In the process of shifting from competition among international enterprises to competition in the supply chain, fourth-party logistics can help enterprises focus on their core business; it integrates capital flow, logistics, business flow and information flow in the global supply chain into a service hub to promote the development of logistics for stakeholders from business supporters to business leaders (22). Fourth-party logistics integrates logistics resources through integration, improves the efficiency of logistics services and optimizes logistics costs to promote the development of the overall logistics service industry. Therefore, fourth-party logistics can offer important support to distribution services in the last mile of cross-border E-businesses in the response to COVID-19.

Fourth-party logistics provides an optimized scheme for third-party logistics, finding the best choice to solve supply chain problems and creating a competitive market advantage together with third-party logistics providers (23). Cross-border E-business will lead to expanded geographic markets (24). Thus, the analysis of interconnections and communications in distribution systems is important for the further development of cross-border E-businesses; in particular, optimizing the structure of the distribution system when connecting cross-border E-business logistics services can help in the future expansion of operations. Internet business increases operational complexity and hence the level of difficulty for companies serving foreign markets (25). Therefore, the application of heuristic technology to the location of distribution centers can further improve the efficiency of cross-border E-business.

Information flows between suppliers and buyers can also have a positive effect on relationship performance, and each party benefits from a reduced cost of operations (26). Thus, it is important to have a distribution system for cross-border E-business that considers the local conditions and transportation environment. Cross-border E-business customers are more dispersed, and the synergies related to distribution and transportation are important for cross-border E-business (27). Additionally, internet shopping delivery is often tightly scheduled, and thus, heuristic solutions to distribution station decisions can help firms to better fulfill the demands of cross-border E-business customers.

The 0–1 programming approach can be helpful in identifying heuristic solutions for the decisions of distribution networks. The 0–1 heuristic model is applied in planning city logistic systems by focusing on the integrated short-term scheduling of operations and the management of resources (28). The 0–1 programming is applied to the integration and consolidation of air cargo shipments (29), while 0–1 programming is applied to find the optimal integration of air cargo shipments based on sequential activities by relevant processing units. This research applies 0–1 programming to analyze the locations of distribution centers in for cross-border E-business, and fuzzy cluster analysis is applied to support the heuristic solutions by considering local conditions. Fuzzy cluster analysis can offer support to study of characteristics of nodes in network structure (30). The heuristic solutions for the distribution network in the gradual expansion of geographic coverage are visualized by a 2-mode network. Artificial intelligence can offer support to analysis of locations in network structure based on inter-connections of nodes (31). The immune algorithm can be further applied to study the key nodes of the network (32). A key node analysis of the distribution network is further performed by immune algorithm, and the expansion of the distribution network from downtown to the suburbs in the last mile of cross-border E-business based on key node analysis is further studied through a simulation based on plant growth. The key node analysis of the distribution network based on the vision of fourth-party logistics can offer further support to business operators for last mile distribution services in smart cities in emerging markets in response to COVID-19. The development of geographic coverage in distribution network is further simulated following organic growth of plant, and the simulation offers further support to understanding in development of geographic coverage of distribution network in response to COVID-19.

## Research Methods

This research analyzed the distribution centers serving cross-border E-business operations in Beijing for a heuristic solution to the location decision for distribution centers based on the vision of fourth-party logistics by integrating resources in the distribution network for the last mile of cross-border E-business. For efficient interconnections between the local distribution system and arriving merchandise shipped to Beijing by air or land, this research analyzed the local conditions of distribution centers by considering the urban environment and transportation facilities. The geographic information of the distribution network is obtained from map.baidu.com. This research analyzes 582 sample distribution centers serving the cross-border E-business area in Beijing city, a smart city in an emerging market, for a case study of key node analysis in response to COVID-19.

This research classifies the distribution centers into different groups by fuzzy cluster analysis based on the urban environment and transportation facilities. The location conditions included in the fuzzy cluster analysis are distributions of neighboring units and areas that could be smart, including a number of connections to science and industrial parks, residential areas, education and research institutes, commercial buildings, shopping centers, parks, sports fields, exhibition centers, and hospitals. The transportation facilities considered in the fuzzy cluster analysis includes the connections between distribution centers and transportation facilities, including the number of connections to highways, loops, and main roads in Beijing. The number of clusters in the fuzzy cluster analysis is identified by the Xie-Beni index (33), which is as follows:


$$V_{XB}=\frac{\sum_{i=1}^{c}\sum_{j=1}^{n}\mu_{ij}{\;}^{2}{\left\|x_{i}-\upsilon_{i}\right\|}^{2}}{n\;\underset{i\neq j}{\min}{\left\|\upsilon_{j}-\upsilon_{i}\right\|}^{2}}$$

where V_XB_ is the Xie-Beni index, X_i_ is the database of local conditions and transportation facilities, and is the vector of centroids. The minimum V_XB_ leads to an optimal number of clusters in the fuzzy cluster analysis. The data involved in the fuzzy cluster analysis are standardized.

The 0–1 LP programming is applied to find a heuristic solution for the location of distribution centers, and it is performed by the MATLAB program. The objective function of the program is to find a heuristic solution to the decision of locating distribution centers that fulfill the specified conditions. The function of the program is as following:


$$\{\begin{array}{l} \min\;f=x_{1}+x_{2}+\cdots x_{582}\\ s.t.\;x_{1ci}+x_{2ci}\cdots x_{582ci}\geq a_{i}\\ \;x_{1compi}+x_{2compi}\cdots x_{582compi}\geq b_{i}\\ \;x_{1disi}+x_{2disi}\cdots x_{582disi}\geq c_{i}\\ \;\alpha_{1}x_{1\text{faci}}+\alpha_{2}x_{2\text{faci}}\cdots \alpha_{3}x_{582\text{faci}}\geq d_{i}\\ \;\beta_{1}x_{1trani}+\beta_{2}x_{2train}\cdots \beta_{3}x_{582train}\geq e_{i}\\ \;x_{i}\in (0,1) \end{array}$$

where min f = x_1_ + x_2_ +…+x_582_ is the objective function that minimizes the total number of distribution centers while fulfilling the follow-up conditions.

1. x_ici_ is the decision of distribution station x_i_ selected from cluster c_i_, and the total number of distribution centers selected from cluster c_i_ is larger than a_i_.
2. x_icompi_ is the decision of distribution station x_i_ selected from express company comp_i_, and the total number of distribution centers selected from comp_i_ is larger than b_i_.
3. x_idisi_ is the decision of distribution x_i_ selected from district_i_, and the total number of districts selected from district_i_ is larger than c_i_. The districts in Beijing include Changping, Chaoyang, Daxing, Dongcheng, Fangshan, Fengtai, Haidian, Huairou, Mentougou, Miyun, Pinggu, Shijingshan, Shunyi, Tongzhou, Xicheng, and Yanqing.
4. x_ifaci_ is the decision of distribution x_i_ selected in connection to facility_i_, and is the number of connections to facilities. The total number of facilities connected to fac_i_ is larger than d_i_. The facilities include a number of connections to highways, loops, and main roads.
5. x_itrani_ is the decision of distribution x_i_ selected in connection to transportation stations, and is the number of connections to transportation station tran_i_. The total number of transportation stations selected in connection to transportation station tran_i_ is larger than e_i_. The transportation station tran_i_ includes the number of connections to highways, loops, and main roads.

The key nodes of the distribution network in the last mile distribution of cross-border E-business in Beijing city are further analyzed by a immune algorithm. The population is important to the management of disease control (34). The identification of key nodes based on the vision of fourth-party logistics can support for solutions in the last mile distribution network of cross-border E-businesses in smart cities in emerging markets in response to COVID-19. The mobility of the population can be important to disease control in different cities (35). Contact in smart cities can further increase the transfer of disease based on surfaces (36). The locations of key nodes in the distribution network are further studied. Extensions in the distribution network are simulated based on a plant growth trends with geographic distribution from the downtown to the suburbs smart cities.

## Results

The distribution centers serving cross-border E-businesses are classified into three clusters based on fuzzy cluster analysis. Table 1 shows the Xie-Beni index for each cluster, and the optimal number of clusters in the fuzzy cluster analysis is found to be 3 clusters, with the minimum Xie-Beni index at 1.1E-13. Table 2 further shows the conditions of 0–1 programming for distribution centers serving cross-border E-businesses in Beijing.

**Table 1:** Xie-Beni Index for Fuzzy Cluster Analysis.

**Cluster**	**C = 2**	**C = 3**	**C = 4**	**C = 5**	**C = 6**	**C = 7**	**C = 8**	**C = 9**
X-B Index	1.65E-13	1.1E-13	4.04E-11	1.6E-13	7.99E-10	6.09E-10	1.13E-10	1.08729
Cluster	C = 10	C = 11	C = 12	C = 13	C = 14	C = 15	C = 16	C = 17
X-B Index	1.26E-07	0.000225	5.18E-07	5.53E-09	0.005077	2.38E-05	4.53E-06	0.000651
Cluster	C = 18	C = 19	C = 20	C = 21	C = 22	C = 23	C = 24	
X-B Index	0.002075	4.76E-05	0.00039	0.053205	3.62E-06	6.3E-07	2.88E-06	

**Table 2:** Conditions for 0–1 Programming of Distribution centers for Cross-border E-business in Beijing.

**Conditions**	**Case**	**C1 (*N* = 25)**	**C2 (*N* = 50)**	**C3 (*N* = 100)**	**C4 (*N* = 200)**	**Weight**
Cluster (a_i_)	C1	8	16	32	65	0.3247
	C2	5	10	20	41	0.2045
	C3	12	24	47	94	0.4708
Express Company (b_i_)	Comp1	6	12	24	47	0.2354
	Comp2	3	7	14	27	0.1357
	Comp3	12	23	47	95	0.4742
	Comp4	4	8	15	30	0.1546
District (c_i_)	CP	2	3	6	12	0.0584
	CY	6	12	24	46	0.2320
	DX	1	2	4	8	0.0395
	DC	2	4	8	15	0.0739
	FS	1	2	4	8	0.0412
	FT	2	5	9	18	0.0911
	HD	4	7	15	30	0.1478
	HR	0	0	1	1	0.0052
	MTG	0	0	1	2	0.0086
	MY	0	1	1	2	0.0120
	PG	0	0	1	2	0.0086
	SJS	1	2	4	9	0.0430
	SY	2	3	5	11	0.0550
	TZ	2	5	10	20	0.0979
	XC	2	4	8	16	0.0825
	YQ	0	0	0	1	0.0034
Facility (d_i_)	Highway	15	29	58	87	0.2906
	Loops	25	50	100	149	0.4980
	Main Road	10	21	42	64	0.2114
	Total	50	100	200	300	
Transport (e_i_)	Airport	8	14	27	55	0.5468
	Railway	7	11	23	45	0.4532
	Total	15	25	50	100	
Optimal Number of Distribution centers		25	50	101	201	

Case 1 plans to select over 25 distribution centers to efficiently serve cross-border E-businesses in Beijing based on the following conditions:

1. The allocation of distribution centers from different clusters follows the weight of the distribution centers in each cluster. Thus, over 8 distribution centers are planned for selection from cluster 1, 5 distribution centers from cluster 2, and 12 distribution centers from cluster 3.
2. The allocation of distribution centers from different express companies follows the weight of distribution centers at each company. Thus, over 6 distribution centers are planned to be selected from express company 1, 3 distribution centers from express company 2, 12 distribution centers from express company 3, and 4 distribution centers from express company 4.
3. The allocation of distribution centers covering different districts follows the weight of distribution centers at each district.
4. The allocation of distribution centers in connection to different facilities follows the weight of connections to each type of facility. Thus, over 15 connections are expected from distribution centers to the highway, over 25 are expected from distribution centers to loops, and over 10 are expected from distribution centers to the main road. Over 50 total connections to facilities are planned.
5. The allocation of distribution centers in connection to different transportation stations also follows the weight of connections to each kind of transportation station. Thus, over 8 connections between distribution centers and airports are expected and over 7 between distribution centers and railways. Over 15 total connections are planned to transportation stations serving cross-border E-businesses.

Case 2 plans to select over 50 distribution centers to efficiently serve cross-border E-businesses in Beijing based on targeting conditions as described in Table 2. Case 3 plans to select over 100 distribution centers, and Case 4 plans to select over 200 distribution centers to serve cross-border E-businesses in Beijing. Table 3 shows the heuristic solutions to the 0–1 programming.

**Table 3:** Heuristic Solutions to 0–1 Programming for Locations of Distribution centers Serving Cross-border E-business in Beijing.

	**Heuristic Solutions to 0–1 Programming of Distribution centers**
Case 1 (Solution = 25)	L2, L8, L9, L11, L12, L50, L153, L168, L203, L221, L233, L323, L346, L356, L361, L390, L404, L426, L439, L450, L482, L525, L538, L542, L581
Case 2 (Solution = 50)	L6, L7, L9, L10, L15, L17, L48, L68, L69, L82, L84, L88, L144, L168, L169, L212, L213, L214, L215, L217, L218, L221, L240, L294, L320, L321, L322, L331, L337, L338, L359, L370, L400, L403, L435, L436, L437, L446, L450, L451, L455, L480, L516, L517, L519, L520, L537, L540, L545, L556
Case 3 (Solution = 101)	L7, L24, L48, L55, L62, L66, L67, L73, L82, L91, L99, L100, L101, L102, L104, L105, L107, L108, L110, L112, L113, L116, L117, L120, L138, L143, L153, L165, L167, L176, L180, L181, L186, L187, L193, L194, L198, L214, L217, L221, L230, L231, L233, L241, L251, L255, L256, L273, L277, L278, L280, L281, L283, L293, L317, L318, L319, L321, L322, L336, L337, L345, L356, L359, L361, L365, L369, L373, L390, L400, L407, L433, L460, L461, L462, L463, L464, L465, L480, L487, L488, L489, L490, L491, L492, L502, L507, L508, L509, L511, L512, L513, L514, L517, L519, L525, L526, L528, L529, L558, L574
Case 4 (Solution = 201)	L1, L3, L9, L48, L49, L55, L63, L64, L66, L68, L82, L92, L93, L94, L95, L96, L97, L98, L99, L100, L102, L104, L105, L106, L107, L108, L110, L111, L112, L113, L114, L115, L116, L117, L118, L119, L120, L121, L122, L123, L130, L131, L133, L134, L135, L136, L137, L152, L156, L184, L185, L186, L187, L188, L189, L190, L191, L192, L193, L194, L195, L196, L197, L198, L199, L200, L201, L202, L203, L207, L210, L212, L214, L216, L228, L229, L230, L231, L233, L234, L235, L237, L238, L239, L240, L241, L242, L244, L247, L248, L251, L252, L255, L256, L257, L258, L259, L260, L261, L266, L267, L268, L269, L273, L277, L278, L280, L281, L283, L289, L290, L291, L292, L293, L294, L297, L298, L299, L311, L312, L314, L315, L316, L317, L318, L319, L320, L321, L332, L333, L334, L335, L336, L337, L339, L340, L344, L345, L348, L349, L350, L351, L352, L353, L354, L355, L356, L357, L358, L361, L363, L378, L399, L400, L418, L420, L421, L436, L437, L438, L439, L484, L486, L487, L488, L489, L490, L491, L492, L494, L497, L499, L500, L502, L503, L504, L509, L510, L511, L512, L513, L514, L515, L516, L518, L519, L520, L525, L529, L539, L556, L557, L558, L566, L567, L568, L569, L572, L573, L574, L576

**Figure 1:**
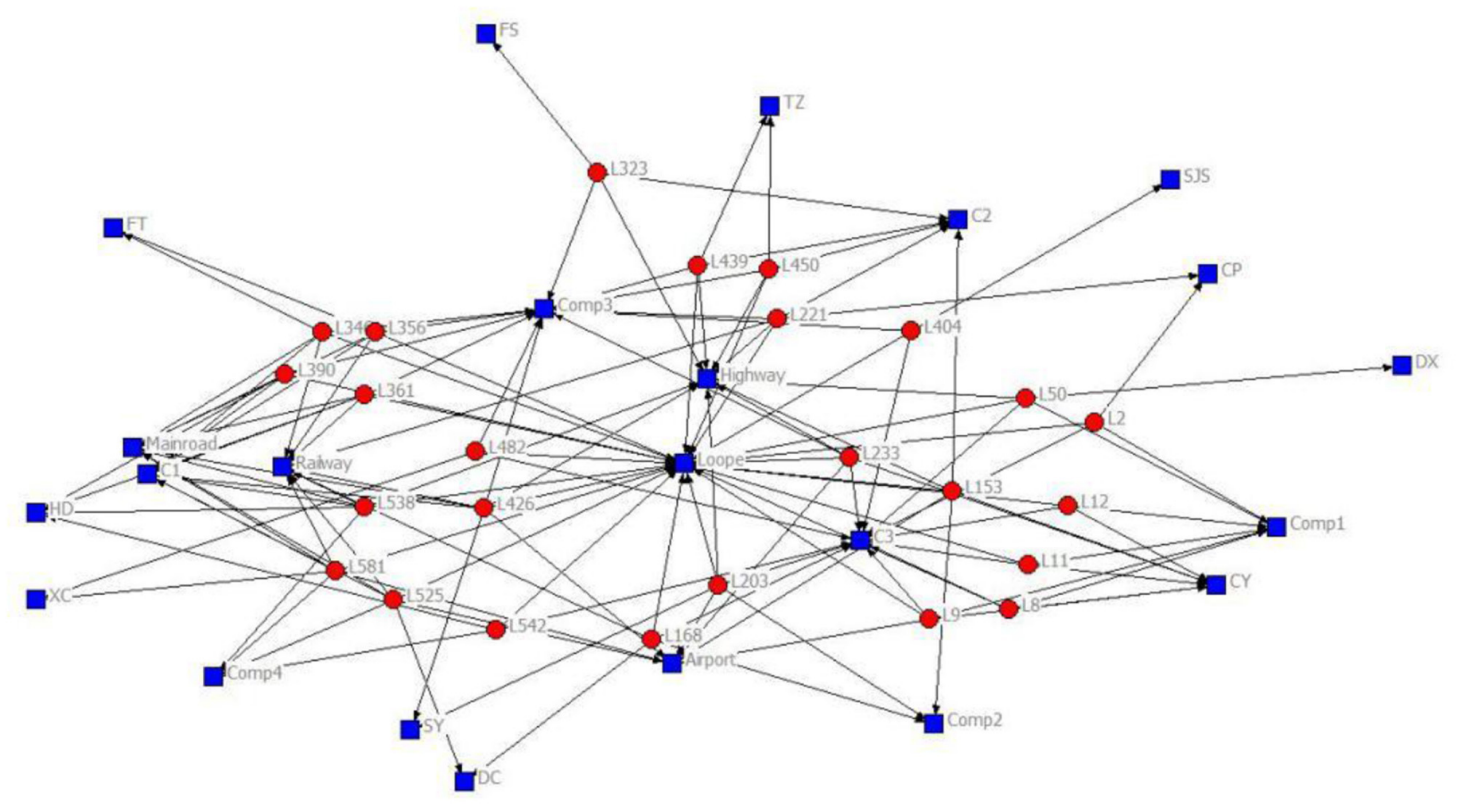
2-Mode Network of Distribution centers and Local Conditions in the Heuristic Solution of Case 1 (Solution = 25).

**Figure 2:**
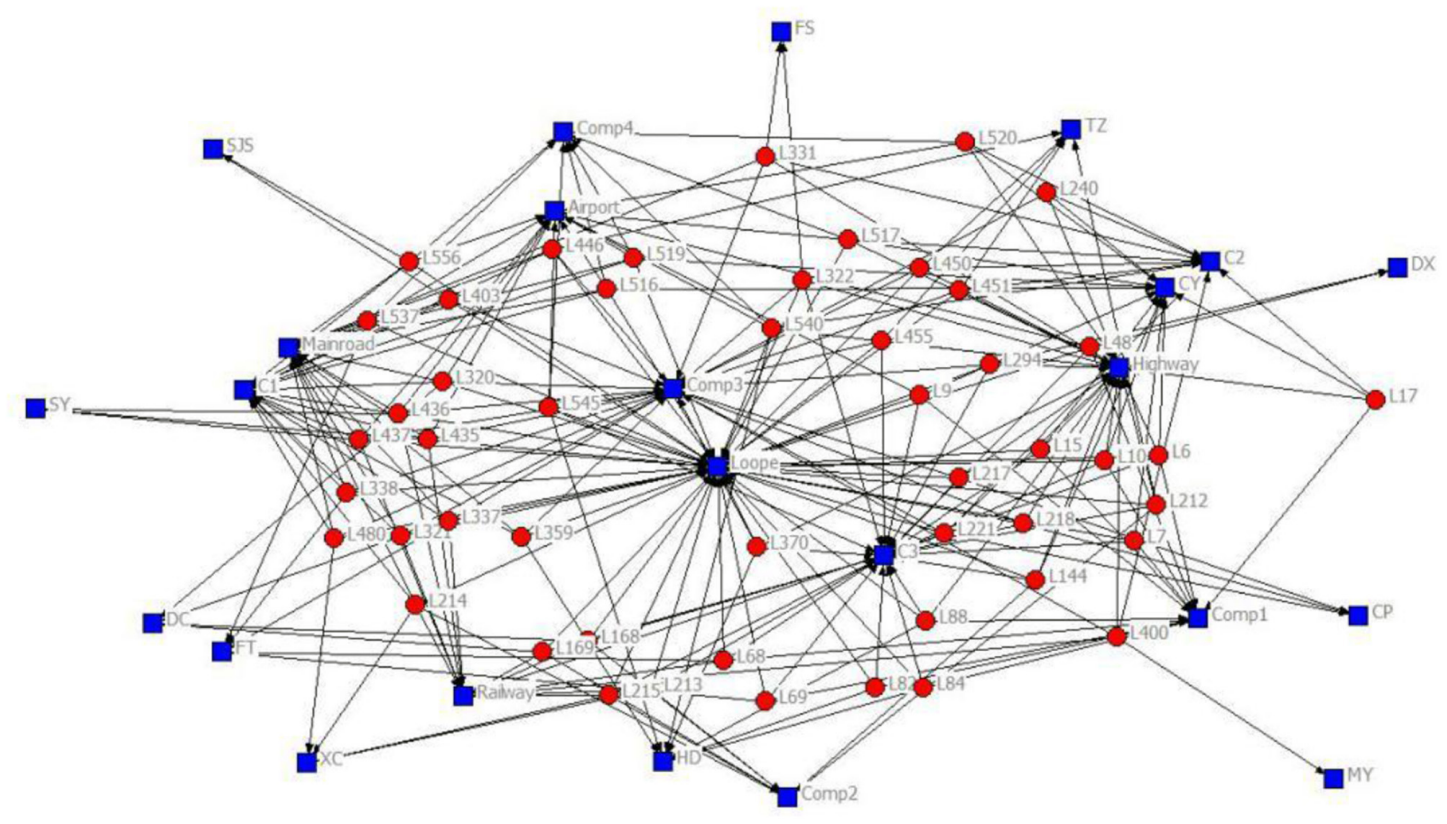
2-Mode Network of Distribution centers and Local Conditions in the Heuristic Solution of Case 2 (Solution = 50).

**Figure 3:**
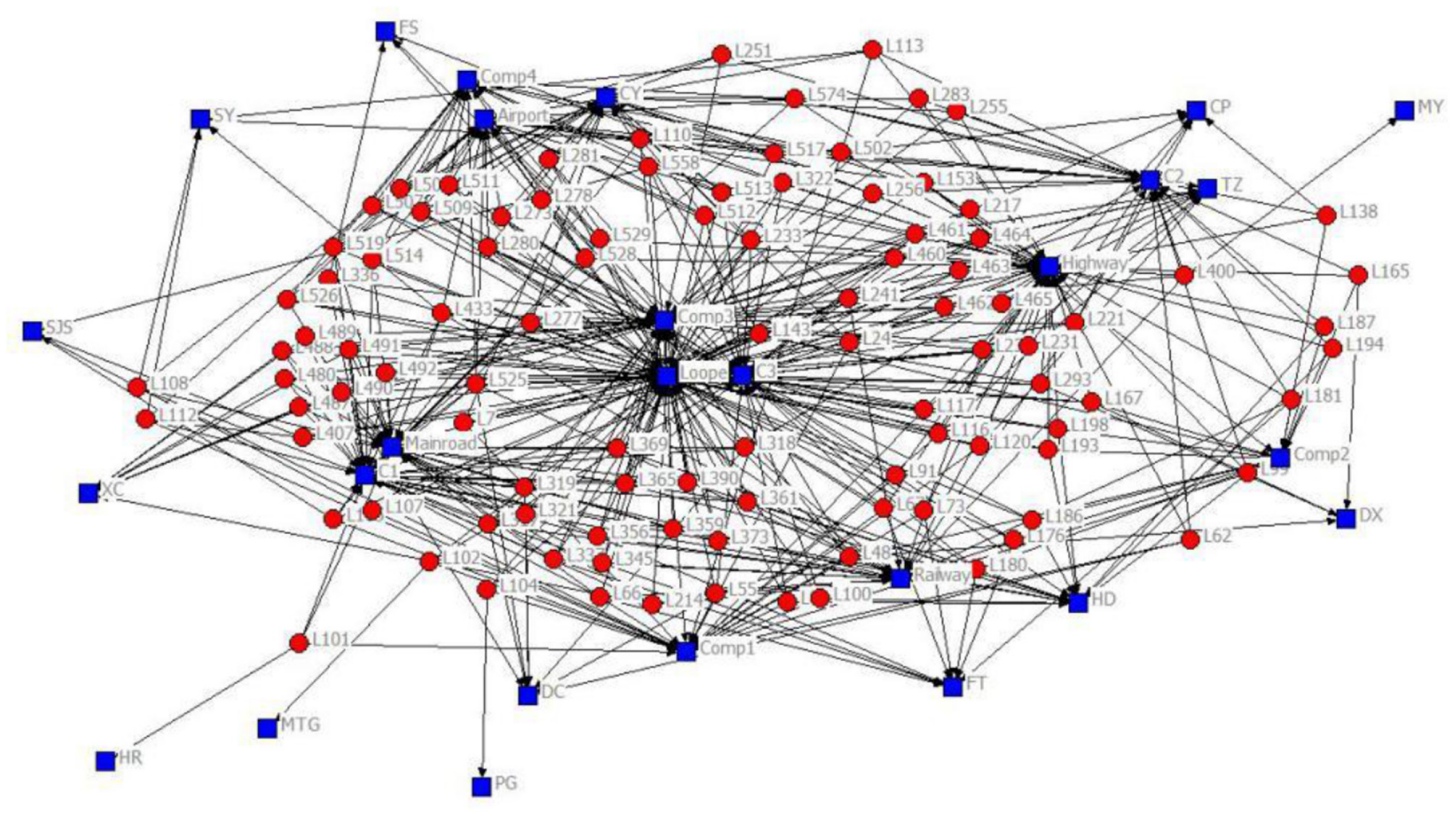
2-Mode Network of Distribution centers and Local Conditions in the Heuristic Solution of Case 3 (Solution = 101).

**Figure 4:**
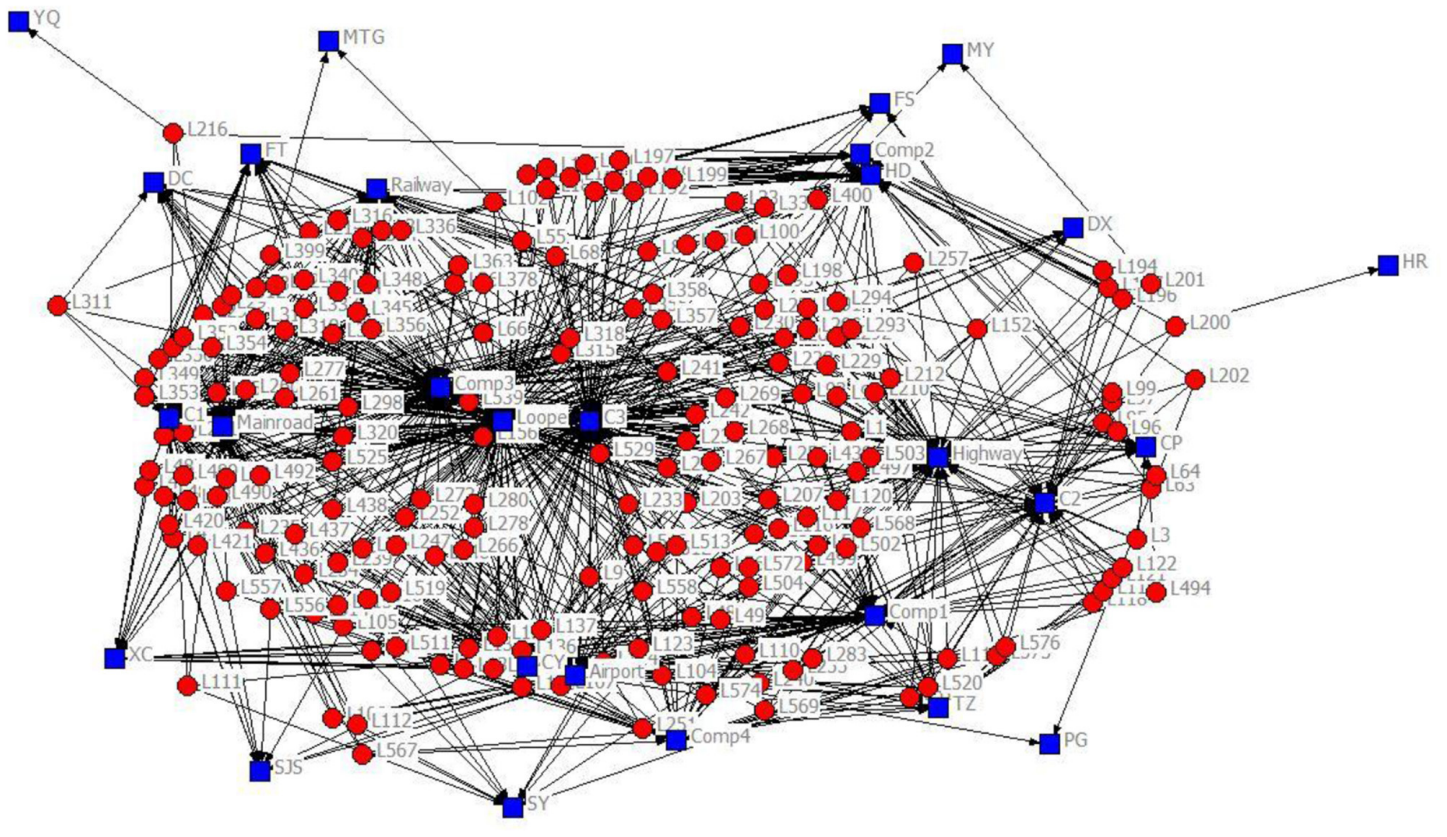
2-Mode Network of Distribution centers and Local Conditions in the Heuristic Solution of Case 4 (Solution = 201).

The 2-mode network of distribution centers and local conditions in the heuristic solution to Case 1 is visualized by Figure 1. The 2-mode network of distribution centers and local conditions in the heuristic solution of Case 2 is visualized by Figure 2. The 2-mode network of distribution centers and local conditions in the heuristic solution of Case 3 is visualized by Figure 3. The 2-mode network of distribution centers and local conditions in the heuristic solution of Case 4 is visualized by Figure 4.

**Figure 5:**
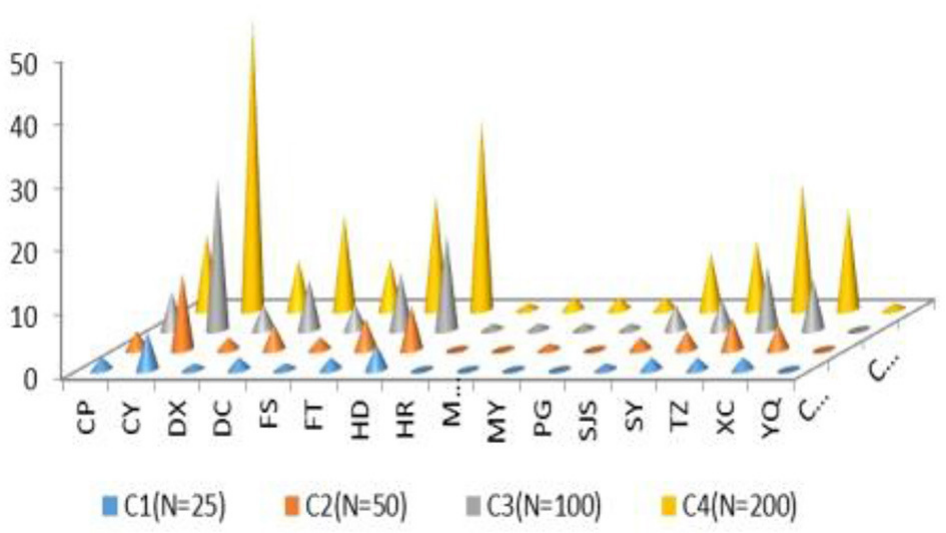
Location of Distribution centers in Network for Last Mile Distribution for Cross-border E-business.

Figure 5 shows the analysis of the distribution network in cross-border E-business value mining. The distribution sites selected from the heuristic solutions are concentrated in Chaoyang, Haidian and Tongzhou Districts. The acceleration of cross-border development and the acceleration of information flow promote the further upgrading of the fourth party logistics value mining mechanism. According to the local traffic conditions in urban areas, the distribution centers are divided into different groups. Based on the analysis of local traffic conditions in urban areas and according to the weight of distribution centers in different clusters, the objective function is used to provide exploratory solutions for the location decision to meet the needs of distribution centers in different situations. Regions with higher weights of distribution centers, such as Chaoyang District, Haidian District and Tongzhou District, contribute more to the solution. These regions have higher demand for last mile distribution services.

Chaoyang District belongs to an area of commercial concentration. The development of its distribution network should be coordinated with the planning of the commercial district to further promote intelligent services and support the development of a dense smart commercial district. Furthermore, Haidian District is an educational location with a concentration of colleges and universities. Its distribution network development should cooperate with academic institutions and provide innovative service modes and systems for college students. Tongzhou District is a dense smart residential area, and the development of its distribution network should be coordinated with the residential area and the service system to provide convenience for the residential population and strengthen communication and cooperation with the local management system. In other words, a system for last mile service and institutional support for the upgrading of the distribution network based on local characteristics can be promoted in the process of value mining for cross-border E-business, and the upgrading of service and value mining can be the driving force for the further upgrading of the system.

**Figure 6:**
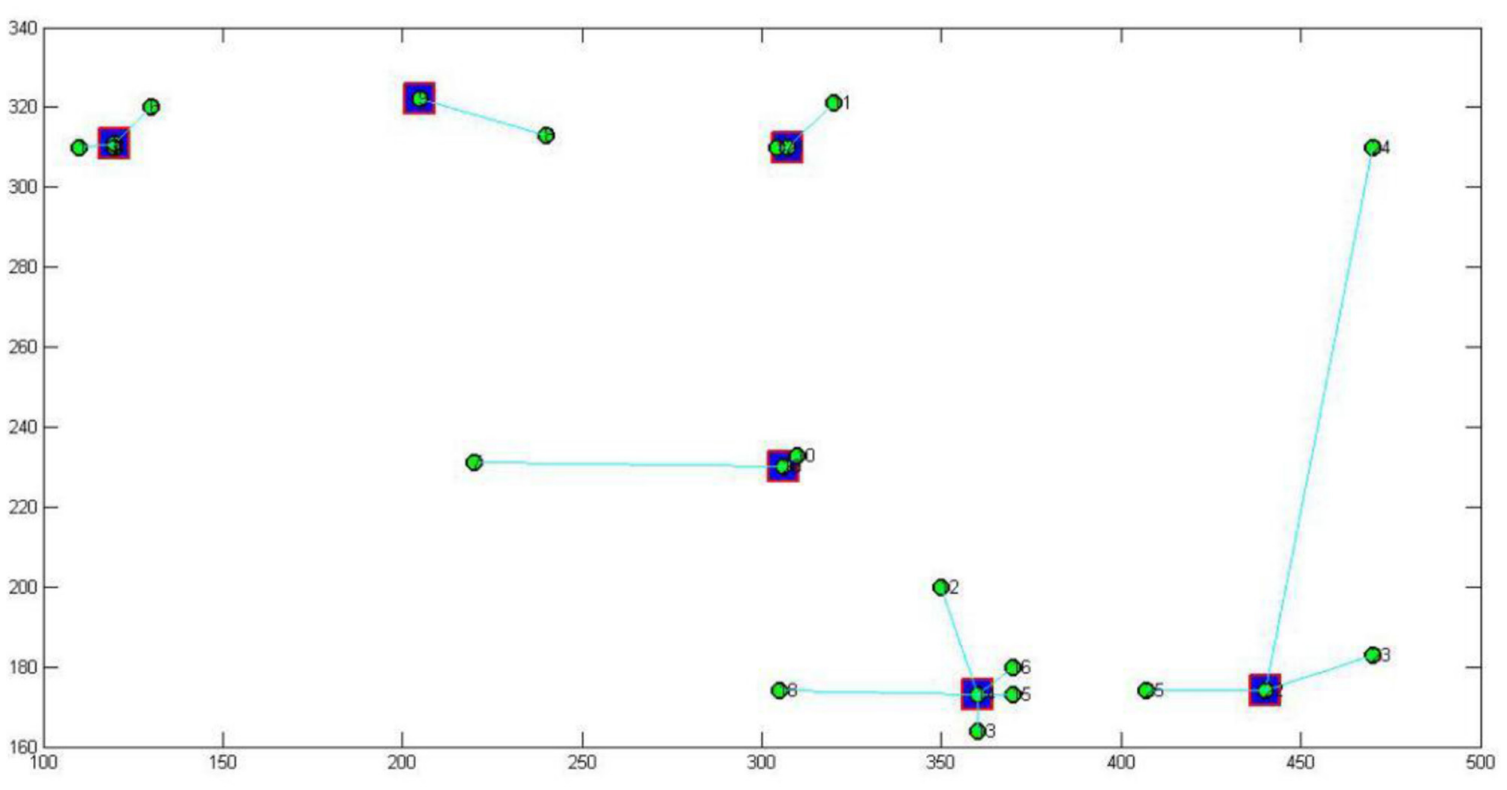
Key Nodes in the Distribution Network of Case 1.

**Figure 7:**
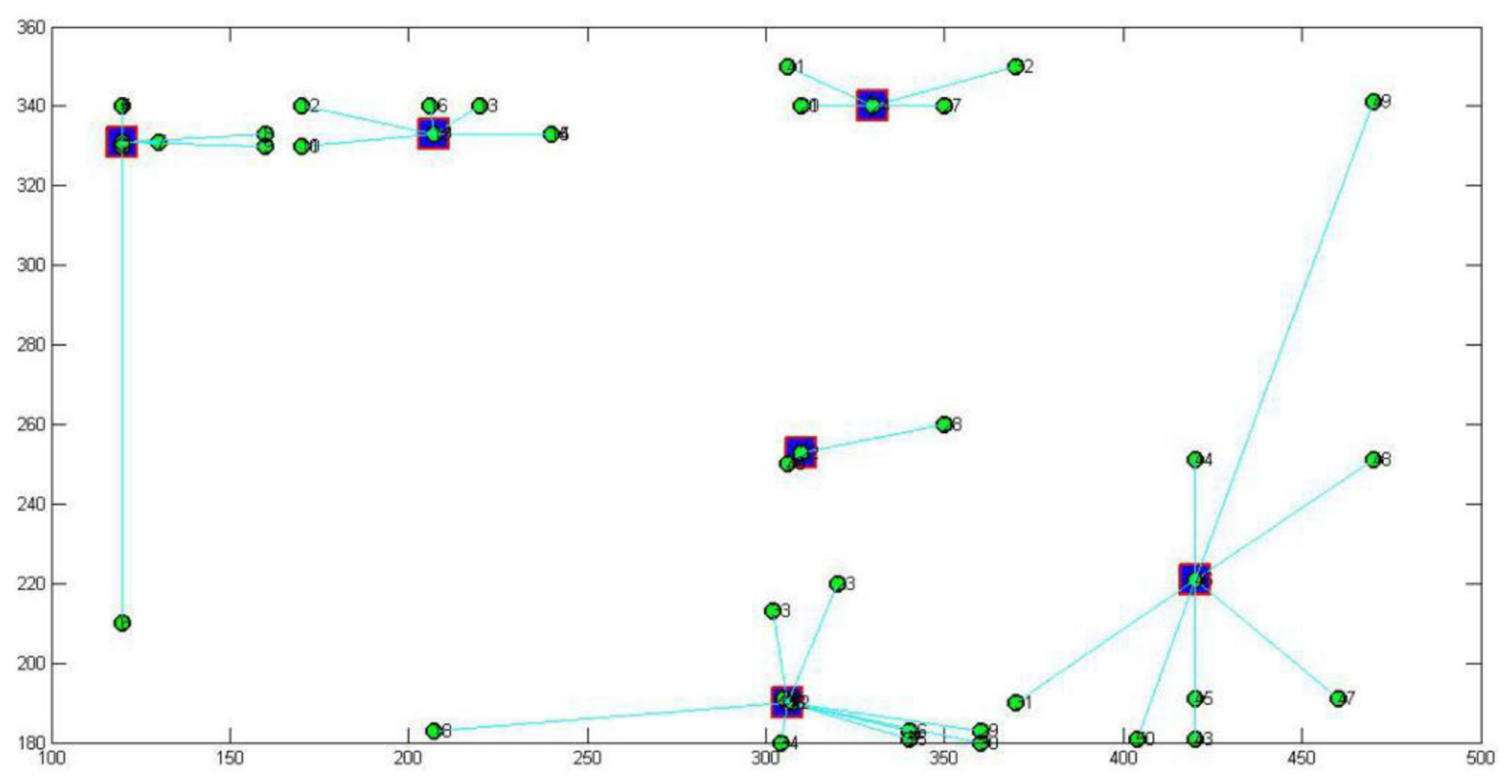
Key Nodes in the Distribution Network of Case 2.

**Figure 8:**
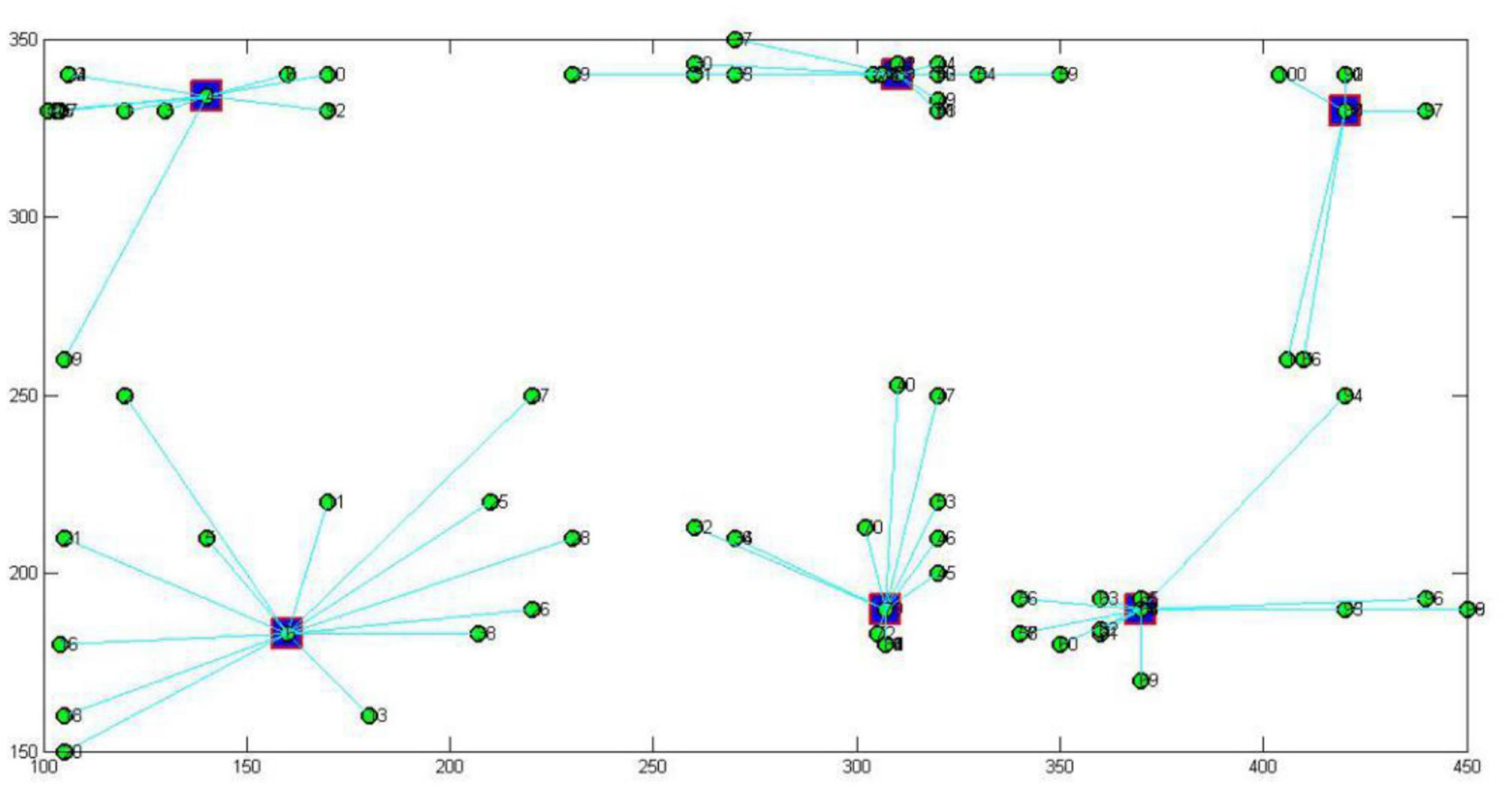
Key Nodes in the Distribution Network of Case 3.

**Figure 9:**
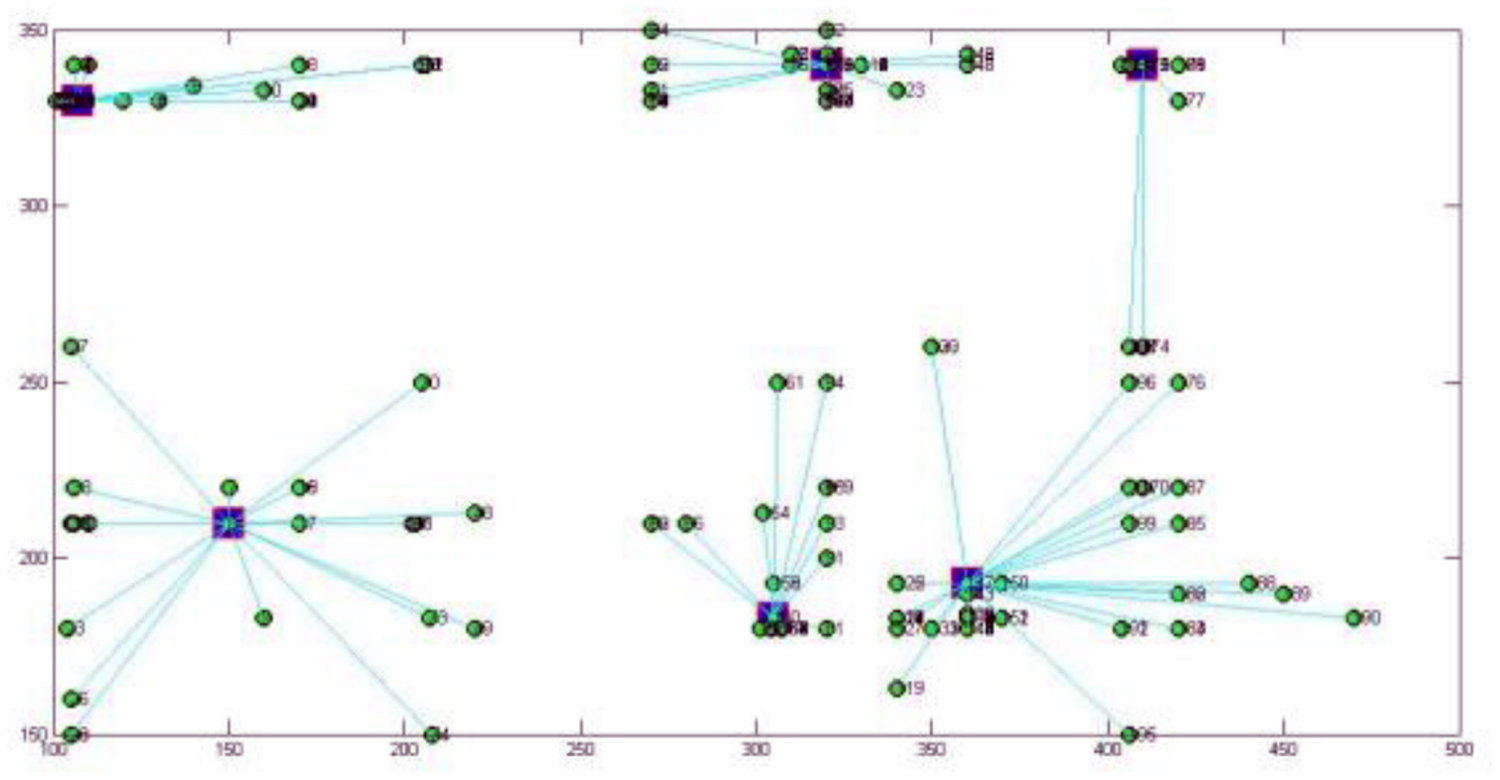
Key Nodes in the Distribution Network of Case 4.

This study further discusses the key nodes of the Beijing cross-border E-business distribution network using a immune algorithm. Figure 6 shows the key nodes in the distribution network of Case 1, Figure 7 shows the key nodes in the distribution network of Case 2, Figure 8 shows the key nodes in the distribution network of Case 3, and Figure 9 shows the key nodes in the distribution network of Case 4. The blue node is the key node in the distribution network.

**Figure 10:**
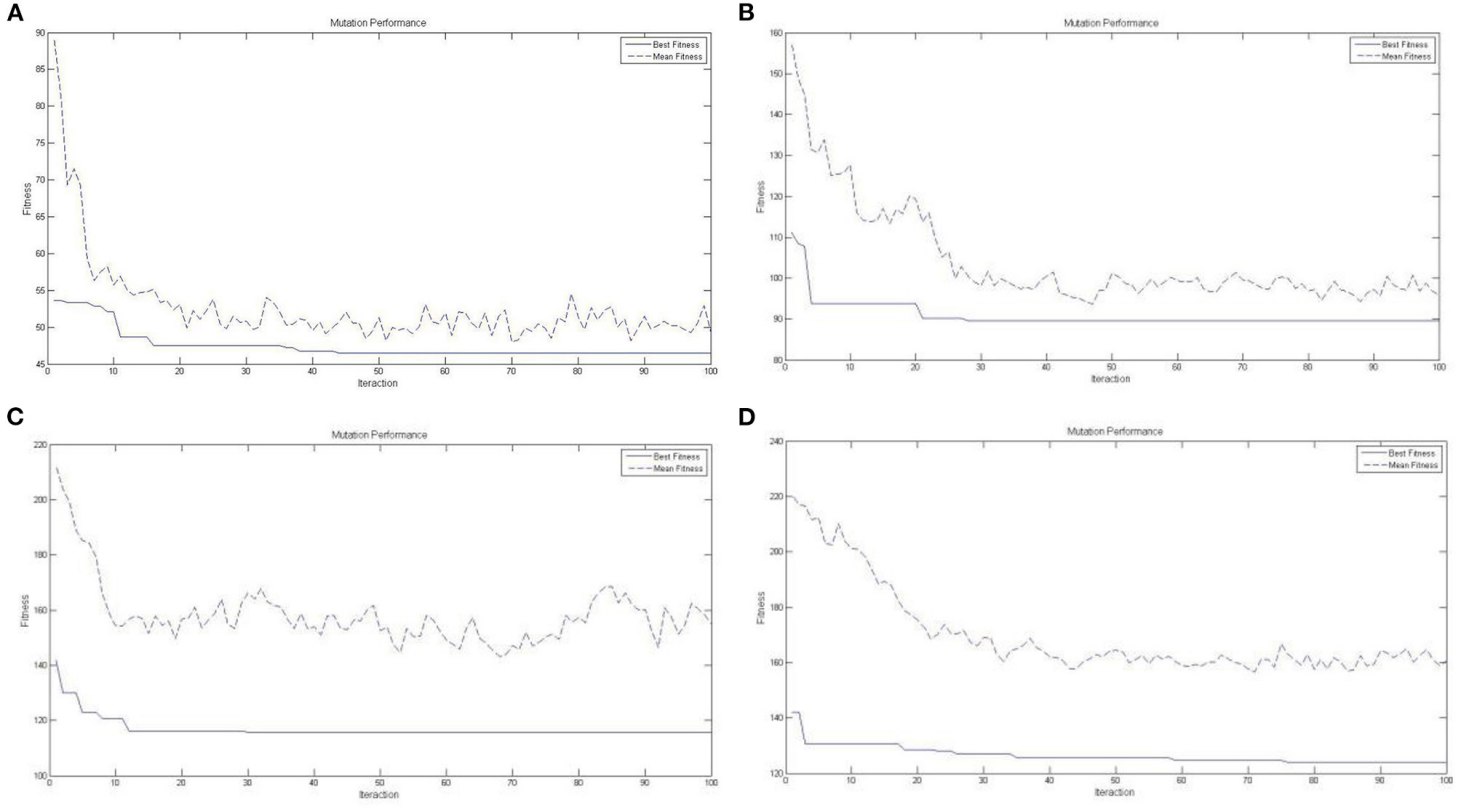
**(A)** Immune Performance for Key Node Analysis based on a Immune Algorithm in Case 1. **(B)** Immune Performance for Key Node Analysis based on a Immune Algorithm in Case 2. **(C)** Immune Performance for Key Node Analysis based on a Immune Algorithm in Case 3. **(D)** Immune Performance for Key Node Analysis based on a Immune Algorithm in Case 4.

Figure 10A shows the performance of the key node analysis based on the immune algorithm in the distribution network of Case 1, Figure 10B shows that based on the immune algorithm in the distribution network of Case 2, Figure 10C shows that based on the immune algorithm in the distribution network of Case 3, and Figure 10D shows that based on the immune algorithm in the distribution network of Case 4. The best fitness assists in the declining trend with mean fitness, and the immune algorithm shows good performance in identifying the key nodes of the distribution network of cross-border E-businesses to further support the last mile distribution network in smart cities in emerging markets in response to COVID-19.

**Figure 11:**
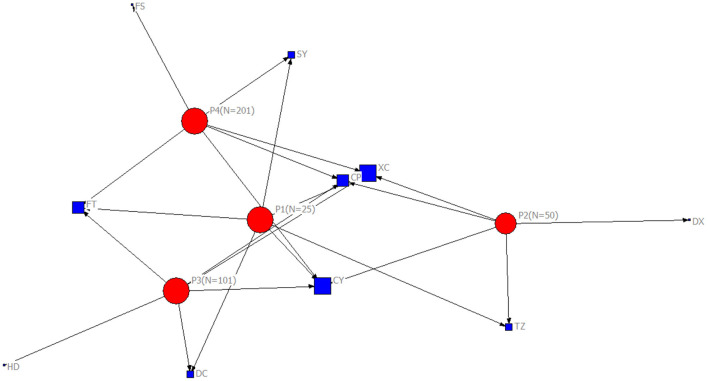
The Location of Key Nodes in the Distribution Network.

Figure 11 shows the distribution of key nodes in Beijing's “last kilometer” distribution service network among regions. With an increase in distribution centers in the distribution network from Case 1 (*n* = 25) to Case 4 (*n* = 201), the network area covered by key nodes becomes more intensive, and the regional distribution covered by key nodes in the distribution network gradually extends from Chaoyang, Xicheng and other urban centers to the surrounding areas, including Changping and Fengtai. In the process of expanding the distribution network, with the increase in the number of stations covered by the network, the key nodes in the surrounding areas of the city gradually establish a connection between the urban center and the surrounding areas of the city and play an important distribution function for the last mile of cross-border E-businesses entering Beijing. The distribution centers at down-town locations can be important to network with concentrated geographic coverage, and the distribution centers at sub-town locations can be further involved in key functions with support to necessary connections for expanded geographic coverage.

**Figure 12:**
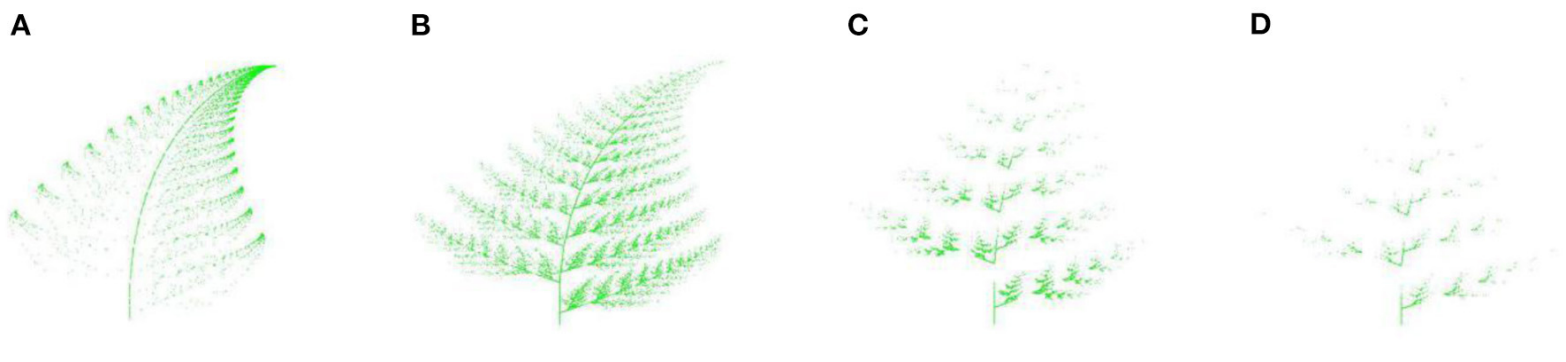
**(A–D)** Simulation of Coverage Area in the Process of Distribution Network Extension.

Figure 12 shows the simulation of the coverage area as the distribution network extends from the central city to the surrounding city. The key nodes in Figure 12A are mainly distributed in the central urban area, and the plants mainly grow in the top green part. The distribution of key nodes in Figure 12B gradually expands from the central urban area to the surrounding areas, and plant growth is distributed in each green area. Figure 12C shows that the distribution of key nodes gradually shifts from the central urban area to the surrounding area, and plant growth is gradually distributed in the green area of the roots. The distribution of key nodes in Figure 12D further spreads across the central urban area and the surrounding connecting areas, and plant growth mainly spreads in the green root area.

## Conclusion

The development of the cross-border E-business market in China encourages companies to further enhance their distribution system networks, as this is required to further improve their efficiency of operations. The application of heuristic technology is important for companies aiming to improve their core competitiveness as they become further involved in the cross-border E-business market. This research analyzed the distribution centers serving the cross-border E-business market in Beijing by fuzzy cluster analysis and considering the local conditions and transportation facilities in Beijing. This research classified the distribution centers into 3 different groups based on the Xie-Beni index. Then, 0–1 programming was applied to identify a heuristic solutions to the location of distribution centers in different cases, targeting a minimum number of distribution centers that fulfill the operational conditions for cross-border E-business logistics in Beijing. The heuristic solution to different cases is further visualized in a 2-mode network. This research applies 0–1 programming to find the heuristic solutions for locating distribution centers and applies fuzzy cluster analysis to analyze their local conditions and transportation facilities. This research can support those looking for heuristic solutions to the location of distribution centers serving cross-border E-businesses in urban areas, and it can help managers and decision makers with the distribution network in the cross-border E-business area.

Furthermore, the key nodes in the Beijing last mile distribution network are analyzed with a immune algorithm. The distribution centers that can further support the development of the cross-border E-business distribution network are concentrated in Chaoyang, Haidian and Tongzhou Districts, and the coordination of the distribution network and system should be further upgraded based on local social and geographical conditions. It is also found that with an increase in the number of distribution centers covered by the key nodes of the distribution network, the location distribution of key nodes will gradually extend from the central urban area to the surrounding areas, and the key nodes will further serve to connect the downtown and suburban areas.

This study can support the location decisions for cross-border E-business distribution centers in the last mile distribution network of smart cities in emerging markets in response to COVID-19. The distribution centers identified by the key node analysis in different cities of the global market can offer important support for maintaining stable supply and distribution services for daily life needs and business operations in the last mile of cross-border E-business in response to COVID-19. The sensible distribution services of cross-border E-businesses, with control of hand-to-hand communication, can also be on trial at key nodes of the distribution network based on unmanned distribution services by locker storage when managing distancing for disease control during COVID-19. The coverage of key nodes in the distribution network also needs to be expanded from downtown to the suburbs as the capacity of distribution in the last mile for cross-border E-business is further extended to help work and production resume in response to COVID-19. In addition, it is important to offer just-in-time confirmation of health conditions for key node distribution locations so that the delivery service is operated in a health-supporting process. The study of key node analysis in distribution network at last mile offers further support to public health management of online-offline service sector in a smart city at emerging markets in response to COVID-19. The key node analysis of the distribution network based on fourth-party logistics can help global managers and decision makers with the last mile distribution network for cross-border E-business in smart cities in emerging markets in response to COVID-19.

## Data Availability Statement

Publicly available datasets were analyzed in this study. This data can be found here: map.baidu.com.

## Author Contributions

DH: conceptualization, resources, data curation, validation, methodology, formal analysis, project administration, writing—original draft, and visualization. XZ: conceptualization, investigation, project administration, and writing—review & editing. YC: conceptualization, investigation, and writing—review & editing. KH: conceptualization, investigation, and writing—review & editing. All authors contributed to the article and approved the submitted version.

## Funding

This research is supported by the National Social Science Foundation of China General Project (20BJL055).

## Conflict of Interest

The authors declare that the research was conducted in the absence of any commercial or financial relationships that could be construed as a potential conflict of interest.

## Publisher's Note

All claims expressed in this article are solely those of the authors and do not necessarily represent those of their affiliated organizations, or those of the publisher, the editors and the reviewers. Any product that may be evaluated in this article, or claim that may be made by its manufacturer, is not guaranteed or endorsed by the publisher.
